# Correction to: Emerging Imaging Modalities in Regenerative Medicine

**DOI:** 10.1007/s40139-018-0171-0

**Published:** 2018-06-05

**Authors:** Mitchel R. Stacy, Albert J. Sinusas

**Affiliations:** 1 Department of Internal Medicine, Yale University School of Medicine, P.O. Box 208017, Dana-3, New Haven, CT 06520 USA; 2 Departments of Internal Medicine & Diagnostic Radiology, Yale University School of Medicine, P.O. Box 208017, Dana-3, New Haven, CT 06520 USA

**Correction to: Curr Pathobiol Rep (2015) 3(1):27–36**

10.1007/s40139-015-0073-3

The article “Emerging Imaging Modalities in Regenerative Medicine,” written by Mitchel R. Stacy and Albert J. Sinusas, was originally published electronically on the publisher’s internet portal (currently SpringerLink) on 28 January 2015 without open access. With the author(s)’ decision to opt for Open Choice, the copyright of the article changed on May 2018 to © The Author(s) 2018 and the article is forthwith distributed under the terms of the Creative Commons Attribution 4.0 International License (http://creativecommons.org/licenses/by/4.0/), which permits use, duplication, adaptation, distribution, and reproduction in any medium or format, as long as you give appropriate credit to the original author(s) and the source, provide a link to the Creative Commons license, and indicate if changes were made.

## Open Access

This article is distributed under the terms of the Creative Commons Attribution 4.0 International License (http://creativecommons.org/licenses/by/4.0/), which permits use, duplication, adaptation, distribution, and reproduction in any medium or format, as long as you give appropriate credit to the original author(s) and the source, provide a link to the Creative Commons license, and indicate if changes were made.

